# Mechanical Thrombectomy in Medium Vessels Occlusion (MeVOs): An Institutional Experience with M2 Divisions of Middle Cerebral Artery

**DOI:** 10.15388/Amed.2024.31.1.18

**Published:** 2024-02-27

**Authors:** Bheru Dan Charan, Shailesh B Gaikwad, Savyasachi Jain, Ajay Garg, Leve Joseph Devarajan Sebastian, M V Padma Srivastava, Rohit Bhatia, Awadh Kishore Pandit, Shashank Sharad Kale

**Affiliations:** 1 Department of Neuroimaging & Interventional Neuroradiology, All India Institute of Medical Sciences, New Delhi, India; 2 Department of Neurology, All India Institute of Medical Sciences, New Delhi, India; 3 Department of Neurosurgery, All India Institute of Medical Sciences, New Delhi, India

**Keywords:** MT mechanical thrombectomy, Ischemic stroke, recanalization, mechaninė MT trobektomija, išeminis insultas, rekanalizacija

## Abstract

**Background:**

Mechanical thrombectomy has been established as a safe, standard and effective treatment option for occlusions of the proximal segment of the middle cerebral artery (MCA), as demonstrated in numerous studies. However, performing thrombectomy in the M2 divisions of MCA presents inherent challenges. In this institutional experience, we aim to delineate the recanalisation rates achieved through mechanical thrombectomy in cases involving the M2 segment of the MCA.

**Methods:**

We conducted a retrospective analysis of patients who underwent thrombectomy due to M2 MCA occlusions in the period from January 2018 to December 2021. Various factors affecting recanalisation rates were assessed.

**Results:**

A total of 15 patients with M2 segment occlusions of the middle cerebral artery were included in the study, comprising 11 in the superior division and 4 in the inferior division. The successful recanalisation rate was 72.33%, with notably higher success observed in cases of inferior division occlusion. The primary outcome of our study was the mTICI recanalisation status, categorised as successful recanalisation (mTICI = 2b or mTICI = 3) and unsuccessful recanalisation (mTICI = 1 or mTICI = 2a) and mRS at 6 months. None of the predictors assessed reached statistical significance.

**Conclusions:**

Mechanical thrombectomy demonstrates favourable efficacy and recanalisation rates in cases of M2 MCA division occlusion. Notably, inferior division occlusions exhibit a higher likelihood of successful recanalisation.

## Abstract (it)

**Įvadas:**

Mechaninė trombektomija, kaip įrodyta daugybe tyrimų, yra saugi, standartinė ir veiksminga vidurinės smegenų arterijos (MCA) proksimalinio segmento okliuzijos gydymo priemonė. Tačiau trombektomijos atlikimas MCA M2 segmente kelia iššūkių. Šia institucie patirtimi siekiame apibrėžti, kiek rekanalizacijos buvo pasiekta atliekant mechaninę trombektomiją MCA M2 segmente.

**Metodai:**

Atlikome retrospektyvinę pacientų, kuriems buvo atlikta trombektomija dėl MCA M2 segmento okliuzijų analizę nuo 2018 m. sausio mėn. iki 2021 m. gruodžio mėn. laikotarpiu. Buvo vertinami įvairūs veiksniai, turintys įtakos rekanalizacijos rodikliams.

**Rezultatai:**

Iš viso į tyrimą įtraukta 15 pacientų, kuriems buvo M2 segmento vidurinės smegenų arterijos okliuzijos, iš jų 11 - viršutinės ir 4 - apatinės dalies. Sėkmingos rekanalizacijos rodiklis buvo 72,33 %, o ypač sėkminga rekanalizacija buvo pastebėta apatinio segmento okliuzijos atvejais. Pirminis mūsų tyrimo rezultatas buvo mTICI rekanalizacijos būklė, skirstoma į sėkmingą rekanalizaciją (mTICI = 2b arba mTICI = 3) ir nesėkmingą rekanalizaciją (mTICI = 1 arba mTICI = 2a) bei mRS po 6 mėnesių. Nė vienas iš įvertintų prognozavimo veiksnių nebuvo statistiškai reikšmingas.

**Išvados:**

Mechaninės trombektomijos veiksmingumas ir rekanalizacijos rodikliai M2 MCA segmento okliuzijos atvejais yra palankūs. Pažymėtina, kad inferiorinių dalių okliuzijos pasižymi didesne sėkmingos rekanalizacijos tikimybe.

## Background

Endovascular mechanical thrombectomy has become a standard treatment modality for ischemic stroke resulting from intracranial large vessel occlusion as described in various multiple randomised control trials. However, it is important to note that there is a paucity of cases involving M2 MCA occlusions in these trials [1]. Treating M2 MCA occlusion with mechanical thrombectomy poses several challenges owing to the tortuous nature of the vessels, the inherent risk of vessel injury, and the potential for subarachnoid haemorrhage (SAH). Interestingly, a limited number of studies have suggested that M2 MCA occlusions may exhibit a more favourable response to intravenous thrombolysis when compared to proximal M1 occlusions [2]. Numerous studies have provided evidence that mechanical thrombectomy (MT) for occlusions in the M2 segment of the middle cerebral artery is a safe and effective intervention, resulting in favourable functional outcomes and a reduced incidence of adverse events [3]. The question of whether mechanical thrombectomy can be employed for M2 branches of the MCA remains a topic with many unanswered questions, as prior evidence suggests that IV tPA alone may suffice for recanalisation and positive outcomes.

New studies have reported that the use of stent retrievers in M2 MCA mechanical thrombectomy is associated with a higher likelihood of successful recanalisation compared to IV-TPA [4, 5].

Our aim in this retrospective study is to analyse recanalisation rates within the M2 MCA divisions and compare endovascular thrombectomy (EVT) recanalisation between superior and inferior division occlusions.

## Methods

### Aim, design and setting of the study

We conducted a retrospective analysis of patients who underwent mechanical thrombectomy in our institute, in the period from January 2018 to December 2021. Our study was approved by the institutional research ethics committee. We analysed patient data from our imaging repository (RIS-PACS) and medical records. Imaging and DSA therapeutic intervention procedures were reviewed by two neuroradiologists.

### Characteristics of patients and Inclusion criteria

1. All patients aged 18 years and older who experienced occlusion of the M2 MCA, whether in the superior or inferior division, underwent mechanical thrombectomy.
2. All patients who presented to our institute within a time frame of 6 hours.
3. We excluded the patients who have concomitant M1 MCA occlusion.
4. We excluded patients having aetiology of ICAD, as during this period we have less number cases, due to COVID-19 era.

We collected comprehensive data, including demographic information, NIHSS (National Institutes of Health Stroke Scale) scores, IV thrombolysis status, ASPECTS (Alberta Stroke Program Early CT Score), recanalisation assessed by TICI (Thrombolysis in Cerebral Ischemia) perfusion grade, and details of emergency treatment. This data was retrieved from our PACS system by querying patient-specific UHID (Unique Health Identifier) and available medical records. The treatment methods employed for achieving recanalisation were categorised and recorded.

### Treatment protocol

At first patient is accessed by a neurologist in the emergency who has symptoms of acute ischemic stroke within the window period of 6 hours. Then we identify M2 MCA vessel occlusion by using CT angiography. After obtaining proper consent with or without IV thrombolysis, the patient was shifted to angiosuite for mechanical thrombectomy.

### NCCT and CT angiography evaluation

NCCT (128 slice CT scanner, Siemens, Erlangen, Germany) was analysed for the presence of hyperdense vessel signs, and ASPECTS score and Triple-phase computed tomography angiography (CTA) images were evaluated for the vessels involved (like the superior or inferior division of M2 MCA) and collaterals scores and to rule out ICAD changes.

### Mechanical Thrombectomy procedure details

**Figure 1:**
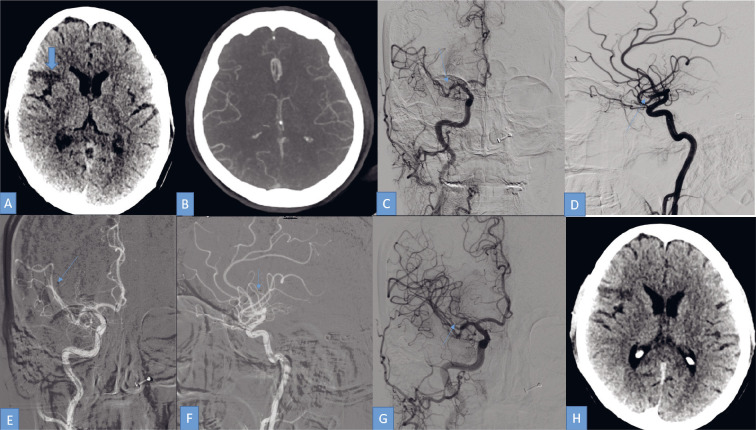
A 42-year patient, a known case of rheumatic heart disease with mitral valve stenosis, presented with acute onset left side weakness and facial deviation for 2 hours. On examinations, her NIHSS was 8 and E4V5M6. IV thrombolysis (rTPA) was given. ASPECTS score was 9 and the collateral was good. On CT angiography there was a cut-off noted in the right M2 MCA superior division. After proper written consent, a mechanical thrombectomy was performed under local anaesthesia. NCCT head axial image at the level of basal ganglia shows cortical hypodensity (arrow) or acute infarct in the right frontal lobe.CT Angio 2nd phase axial MPR images show good collaterals.Right carotid artery digital subtraction angiography AP view shows right M2 MCA superior division (arrow) cut off with normal visualisation of inferior division.Right carotid artery digital subtraction angiography lateral view shows right M2 MCA superior division cut off (arrow) with normal visualisation of inferior division.Micro-catheter (trevo trak 21) was placed in the right M2 MCA superior division (arrow indicated micro catheter’s distal tip).A stent retriever (trevo pro 3x36 mm) (arrow indicated distal triple marker of stent retriever) was deployed via microcatheter across the clot and a good opening of the stent retriever was noted.After 1st stent retriever passes, check angio of the right ICA shows complete recanalisation of occluded vessels. (Modified TICI 3 recanalisation.)Post-embo NCCT shows no SAH or ICH with nonprogression of the infarct.

**Figure 2:**
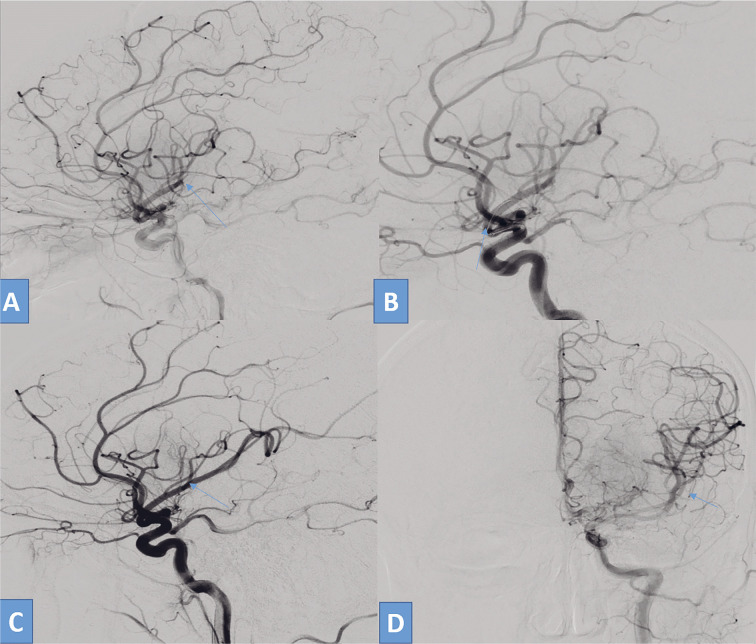
A 48-year-old patient with a history of mitral valve replacement, presented with acute ischemic stroke for 3 hours. She was E4V5M6 with NIHSS 10 with aphasia. NCCT ASPECTS was 9 with good collaterals. Mechanical thrombectomy was done under local anaesthesia. Left ICA DSA lateral view shows cut off (arrow) in the distal segment of M2 MCA inferior division.Microwire and aspiration catheter (3 Max penumbra) assembly.After a single aspiration ADAPT, the check run shows a complete opening of the inferior division (arrow).AP view of left ICA angiogram shows recanalisation of the inferior division of M2 MCA with slow distal flow. (Modified TICI 2b recanalisation.)

MT was performed either solely by the stent retriever technique (SR) (Fig 1), the aspiration technique (Fig 2), or a combination of the two (SA, Solumbra technique). DSA (Biplane DSA, Philips, Netherlands) guided MT performed by three independent neuro-interventional radiologists with experience of more than 10 years. The procedure was performed under local anaesthesia, and general anaesthesia was used for those who were in altered sensorium and did not cooperate locally. Vascular access was obtained using an 8F Short Vascular Sheath. ICA access was subsequently obtained using a Long Sheath and an Intermediate Catheter. Mechanical Thrombectomy was performed using Stent Retriever (Trevo (4x20 and 3x20), Stryker; Solitare (4X40mm), Medtronic) and Aspiration (ACE Reperfusion Catheter, Penumbra). Many cases were converted to the Solumbra procedure after failing the initial stand-alone Stent Retriever/Aspiration thrombectomy.

### Outcome

The primary outcome of our study was mTICI recanalisation status, which we divided into two arbitrary groups: successful recanalisation (mTICI 2b, mTICI 3) and unsuccessful recanalisation (mTICI1, mTICI2a). The modified Rankin scale (mRS) was evaluated after 6 months.

### Statistical analysis

Data were presented as mean and standard deviation for continuous variables and as percentages for categorical variables. An unpaired t-test was done to compare two group means and a paired t-test for paired means within the group. The chi-square test was done to find the association between categorical variables and the Fisher exact test was done if the expected cell count was less than 5. A p-value of less than 0.05 was considered significant.

## Results

In our database, after exclusion, we identified 15 patients who presented with acute ischemic stroke due to M2 MCA branch occlusion and subsequently underwent mechanical thrombectomy at our department. The mean age of the study population, which included 8 males and 7 females, was 50 years.

In our study, we observed a successful recanalisation rate of 72.33% for this medium vessel occlusion (M2 MCA) stroke, defined as achieving mTICI2b or mTICI3 recanalisation, while the unsuccessful recanalisation rate was 26.67%, regardless of the thrombectomy modality used (see Table 1). Among the total of 15 patients, 11 had superior division occlusion, and 4 had inferior division occlusion. Four patients required general anaesthesia for the procedure. Nearly 11 patients were treated with a stent retriever only (Fig 1), three patients underwent the ADAPT technique using 3 MAX (Fig 2), and one patient was treated with the Solumbra technique. Out of 4 unsuccessful recanalised cases, 3 cases were of rheumatic heart disease. In 11 successful cases, 3 were of rheumatic heart disease. All patients were diabetic and hypertensive. 5 have a history of smoking. We conducted a comparison of multiple variables to identify factors contributing to either successful or unsuccessful recanalisation. The significance of these factors was determined based on their p-values. Notably, our analysis revealed that there was no statistically significant correlation between the two groups concerning the patient’s gender, the vessel involved (superior or inferior division), the type of anaesthesia employed, baseline NIHSS, or ASPECTS score. However, it’s worth mentioning that the successful recanalisation rate appeared to be higher in females, cases of inferior division occlusion, procedures performed under general anaesthesia, patients with a baseline NIHSS score greater than 10, and those who received pre-thrombectomy intravenous thrombolysis. However, our results were statistically insignificant. Although we represented our results.

**Table 1:** Comparison of Patient Characteristics and Recanalisation Rates in M2 MCA occlusion stroke.

Variable		Successful (73.33%)	Unsuccessful (26.67%)	P Value
Gender	Male (8)	5	3	0.569
	Female (7)	6	1	
Vessels Involved	Superior (11)	7	4	0.275
	Inferior (4)	4	0	
Anesthesia	LA (11)	7	4	0.275
	GA (4)	4	0	
NIHSS	<10	4	3	0.285
	>/=10	7	1	
Treatment Method: Aspiration	Yes (3)	2	1	1.0
Treatment Method: Stent Retriever	Yes (11)	8	3	1.0
Treatment Method: Solumbra	Yes (1)	1	0	1.0
Baseline ASPECTS	<7	2	1	1.0
	>/=7	9	3	
Pre-EVT IV Thrombolysis	Yes (11)	9	2	0.517

Follow up: Out of our 11 successful recanalised cases, 8 have 6-month mRS<=3 and 3 have mRS>3 in which two have a recurrence of stroke and 1 developed haemorrhage. So mechanical thrombectomy in M2 MCA divisions have a good outcome.

## Discussion

Stroke is a global health issue and ranks as the second most common cause of death and the fourth most common cause of disability worldwide [6]. Timely recanalisation of occluded vessels can prevent patients from experiencing morbidity. The incidence of M2 occlusions may be as high as 7 per 10,000 people per year, with an annual incidence of up to 21,176 in the United States. Nearly 50% of patients with untreated M2 occlusions experience moderate to severe disability at discharge [4]. The available literature regarding the effectiveness of mechanical thrombectomy for acute M2 occlusion has primarily compromised retrospective studies.

The use of the Blind Exchange/Mini-Pinning Technique (BEMP) for medium vessel occlusion in acute ischemic stroke is a safe and effective approach. It is associated with a higher rate of first-pass eTICI 2c/3 recanalisation (66% versus 46%, P=0.037) when compared to using a mini stent retriever alone, as described in a study conducted by Garcia PG et al. [7]. This result is consistent with findings in a study by Hussein et al. [8].

A study by Sarraj et al. [9] reveals that the mechanical thrombectomy group had a 3.1 times greater chance of a favourable outcome compared to standard management with IV TPA. Our study also reported a higher rate of successful recanalisation in M2 occlusions (73.33%) compared to combined ICA, MCA, and basilar artery mechanical thrombectomy (64%). Similar results have been reported in various clinical trials and studies [10, 11, 12, 13].

Kim et al. [14] reported a higher rate of successful recanalisation in M2 occlusions compared to M1 occlusions due to a higher rate of first-pass recanalisation in M2 occlusion.

The reason for the higher first-pass recanalisation rate in M2 MCA is explained by the smaller diameter of the M2 arteries, which enhances clot entrapment by the stent, reduces the dead space between the aspiration catheter and the artery wall, and separates the clot from the artery, thereby increasing suction force. Furthermore, smaller clot sizes in M2 MCA branches are likely more amenable to retrieval.

In our study, the recanalisation rate was higher in inferior division occlusion compared to superior division occlusion. A possible explanation is that superior divisions are usually more anatomically angulated, making them more susceptible to stent-retriever manoeuvres. Occlusion of the superior division was identified as a predictor of poor outcomes, as reported by Seker et al. [15], possibly due to the superior division supplying the central and precentral eloquent areas [16]. Although the number of cases of superior division occlusion was higher, likely because they are more readily identifiable in clinical settings, possibly owing to their association with the motor cortex, our result, similar to another study [17, 18], was not statistically significant.

## Complications

As described in the literature, there is an increased rate of subarachnoid haemorrhage (SAH) in M2 thrombectomy due to vessel tortuosity and small perforators arising from M2 [17]. Therefore, we positioned our aspiration catheter and retriever in M2, avoiding placement in M3 branches. In our study, out of 11 successful cases, 2 have developed foal sulcal sub-arachnoid haemorrhage and none required craniotomy.

## Limitations

There are several limitations to our study. These limitations include patient selection bias attributable to the retrospective nature of our study, its single-centre design, the absence of clear guidelines for selecting patients with AIS secondary to M2 occlusion, and a limited number of patients due COVID-19 era and exclusion criteria. We did not have a control group.

The limited total number of patients in our study may have contributed to the lack of statistical significance in certain variables.

## Conclusions

In summary, our study suggests that mechanical thrombectomy demonstrates favourable outcomes and a high rate of recanalisation in cases of M2 MCA division occlusion. Notably, we observed a greater recanalisation in cases of inferior division occlusion. However, it is imperative to underscore the need for further prospective multicentre studies involving larger patient cohorts to validate our findings, identify prognostic factors, and establish optimal thrombectomy strategies for M2 occlusions.

## Availability of data and material (data transparency)

Yes, on request.
